# The changing epidemiology of opioid overdose in Baltimore, Maryland, 2012–2017: insights from emergency medical services

**DOI:** 10.1080/07853890.2022.2079149

**Published:** 2022-07-01

**Authors:** Chen Dun, Sean T. Allen, Carl Latkin, Amy Knowlton, Brian W. Weir

**Affiliations:** a Department of Surgery, Johns Hopkins University School of Medicine, Baltimore, MD, USA; b Department of Health, Behavior & Society, Johns Hopkins Bloomberg School of Public Health, Baltimore, MD, USA

**Keywords:** Emergency medical services, opioid overdose, naloxone, epidemiology

## Abstract

**Introduction:**

An estimated 100,306 people died from an overdose from May 2020 to April 2021. Emergency Medical Services (EMS) are often the first responder to opioid overdose, and EMS encounter records can provide granular epidemiologic data on opioid overdose. This study describes the demographic, temporal, and geographic epidemiology of suspected opioid overdose in Baltimore City using data from Baltimore City Fire Department EMS encounters with the administration of the opioid antagonist naloxone.

**Method:**

The present analyses used patient encounter data from 2012 to 2017 from the Baltimore City Fire Department, the city’s primary provider of EMS services. The analytic sample included patient encounters within the city that involved naloxone administration to patients 15 years of age or older (*n* = 20,592). Negative binomial regression was used to calculate the incidence rates based on demographic characteristics, year, and census tract. Choropleth maps were used to show the geographic distribution of overdose incidence across census tracts in 2013, 2015, and 2017.

**Results:**

From 2012 to 2017, the annual number of EMS encounters with naloxone administrations approximately doubled every 2 years, and the temporal pattern of naloxone administration was similar to the pattern of fatal opioid-related overdoses. For most census tracts, incidence rates significantly increased over time. Population-based incidence of naloxone administration varied significantly by socio-demographic characteristics. Males, non-whites, and those 25–69 years of age had the highest incidence rates.

**Conclusion:**

The incidence of naloxone administration increased dramatically over the study period. Despite significant cross-sectional variation in incidence across demographically and geographically defined groups, there were significant proportional increases in incidence rates, consistent with fatal overdose rates over the period. This study demonstrated the value of EMS data for understanding the local epidemiology of opioid-related overdose.

**Key Messages**
- Patterns of EMS encounters with naloxone administration appear to be an excellent proxy for patterns of opioid-related overdoses based on the consistency of fatal overdose rates over time.
- EMS plays a central role in preventing fatal opioid-related overdoses through the administration of naloxone, provision of other emergency services, and transportation to medical facilities.
- EMS encounters with naloxone administration could also be used to evaluate the impact of overdose prevention interventions and public health services.

## Introduction

The Centres for Disease Control and Prevention (CDC) reported a 1040% increase in opioid overdose in the United States from 2013 to 2019 [1]. Opioid overdose is increasing amongst the U.S. population and is a leading cause of death, with an estimated 100,306 people have died from an overdose from May 2020 to April 2021 [2]. The number of people dying from an opioid overdose is rising in most states, due in part to increasing fentanyl use, affecting both men and women in all age groups [3,4]. Surveillance data show that intoxication deaths in the City of Baltimore are predominantly opioid-related, with 851 of 914 (93%) intoxication deaths involving opioids, although non-opioids are also commonly present [5].

Through identifying and describing changes in the epidemiology of opioid overdose, surveillance plays an essential role in informing prevention and treatment strategies. The dearth of local data is a major issue for surveillance of opioid overdose, making it difficult for local policymakers to alter policies and respond to changes in epidemics in a timely manner [6–11]. Hospital emergency department records and death records are two common surveillance methods for local opioid overdose epidemiology. Vital statistics data from the CDC [12] is a publicly accessible source of death records, but sample sizes are small for smaller jurisdictions, and lags in data accessibility a year or longer are common [13]. As a result, local researchers and policymakers cannot get accurate information on recent local trends from vital statistics data alone. Hospital emergency departments can provide important local data. However, administrative regions of interest to policymakers, such as city or county boundaries, may include the catchment areas of multiple hospitals, and separate electronic medical records and reporting systems may make hospital-based surveillance challenging.

Emergency Medical Service (EMS) data can be used to provide timely local data on opioid-related overdose [14]. EMS medicks are often the first responders to opioid overdoses, and records of naloxone administration are a useful indicator of suspected opioid-related overdoses. Furthermore, electronic medical records may include geocoded locations (longitude and latitude), and researchers can combine the geocoded locations into census information and incorporate information on patient characteristics and conditions. EMS records can also be used for sentinel surveillance, as EMS patient encounter data are often recorded during or soon after an encounter [10,15]. Indeed, the Council for State and Territorial Epidemiologists has recommended using EMS encounter data among other data systems for surveillance of nonfatal opioid overdoses [16].

Several prior research studies used EMS data as a surveillance method to describe the epidemiology of opioid overdose. Merchant et al. used EMS administration of naloxone and other EMS data to estimate suspected opioid overdoses in Rhode Island and demonstrated that EMS data might be useful for opioid overdose surveillance [17]. Knowlton et al. explored temporal patterns of naloxone administration from Baltimore City EMS data and found that late afternoon, summertime, and the weekend (Friday and Saturday) had the highest incidence rates of suspected opioid overdose [10]. Madah-Amiri et al. found that peak incidence of non-fatal opioid overdoses occurred in August and among males using EMS in Norway [18].

The aims of the present study are to illustrate approaches to analysing EMS data, to describe the demographic and temporal-spatial epidemiology of opioid-related overdose in Baltimore City [19] and to discuss implications for opioid overdose policy and prevention. We combine Baltimore City EMS data and US Census demographic data for the City of Baltimore and its census tracts to identify differences in the population-based incidence of EMS encounters with naloxone administration across census tracts and across groups defined by sex, age, and race/ethnicity. We also describe how the increasing burden of opioid-related overdose, as reflected by EMS naloxone administration, is disproportionately borne by different demographic groups.

## Methods

### EMS data

The data were collected through the BQUEST (Baltimore Quality Urban Emergency Services and Treatment) study [10], approved by the Johns Hopkins University Bloomberg School of Public Health’s Institutional Review Board (IRB number 00002092). We used EMS data obtained from the Baltimore City Fire Department and included EMS encounters from January 2012 to December 2017 in which naloxone was administered. For the analytic sample, we excluded EMS encounters occurring outside the city boundary and encounters among patients younger than 15 years of age, with the latter exclusion used to avoid large low-risk age groups skewing population-based incidence rates. Data from encounters included the date, time, geocoordinates of patient encounter demographics, vitals, medick assessments, medical procedures, medications administered, change in patient condition, patient disposition, transportation, and destination. Multiple non-fatal overdose encounters with the same person were treated as separate encounters and due to data restrictions for patient confidentiality, we did not identify how many individuals had one or more EMS encounters with naloxone administration. Other research indicates that repeat encounters are common, even during relatively short periods of time [20].

Data on medications included dosage, route of administration, and participant response to medication (improved, worse, or unchanged). Demographics included sex (male or female), race (White, Black or African American, American Indian or Alaska Native, Asian, Native Hawaiian or Pacific Islander, other, or unknown), ethnicity (Hispanic or Latino or not Hispanic or Latino), and age (in units of years, months, or days). We recorded demographic variables to match categories used in the US Census so that population-based rates could be calculated. Race and ethnicity were categorised into non-Hispanic White, non-Hispanic Black, and Hispanic of any race or other, and age was categorised into 5-year age groups from 15–19 to 80–84, and 85 or older.

EMS encounters were spatially joined to Baltimore City census tracts using ArcGIS Pro, with shapefiles obtained from the US Census Bureau [21,22]. Population estimates by sex, race, ethnicity, age, and census tract in Baltimore City were from the 2010 U.S. Census [23], with the decennial census providing a greater demographic breakdown than the yearly American Community Survey. To provide population-based denominators for EMS data, census data on race and ethnicity were grouped as non-Hispanic White, non-Hispanic Black, and Hispanic/other, and age included 5-year age groups from 15–29 to 80–84, and 85 or older.

### Statistical analyses

The first analysis described the city-wide pattern of EMS encounters with naloxone administration per 1000 person-years per month. The numerator included all encounters in the analytic sample regardless of missing data on sex or race/ethnicity. The denominator for the rate included all city residents 15 years of age or older and accounted for the number of days in each month. These rates were presented as a time series along with a time series of the annual number of fatal opioid-related overdoses per year within Baltimore City to allow a visual comparison of the consistency across these measures with respect to time [24].

To describe the spatial and spatial-temporal patterns of EMS naloxone administration over time, we calculated the yearly incidence rate for each census tract in Baltimore City. ArcGIS was used to produce choropleth incidence maps for Baltimore City for the years 2013, 2015, and 2017.

We also calculated city-wide yearly incidence rates by sex, race/ethnicity, and age group, and the numerators excluded encounters with missing data on the relevant demographic measure. Demographic incidence rates were presented as time-series graphs. We used negative binomial regression to calculate incidence rates and 95% confidence intervals for each demographic characteristic for the years 2013, 2015, and 2017, and to evaluate whether the incidence rates varied across demographic characteristics. We also estimated the incidence rate ratios by comparing the incidence in 2015 to 2013 and 2017 to 2015 for each demographic characteristic, and we used interaction terms to evaluate significant variation in the IRRs by year across demographic characteristics. Statistical analysis in this study was performed in Stata version 14. All statistical tests were 2-sided, and the level of significance was set at *p* < .05.

## Results

**Figure 1.**
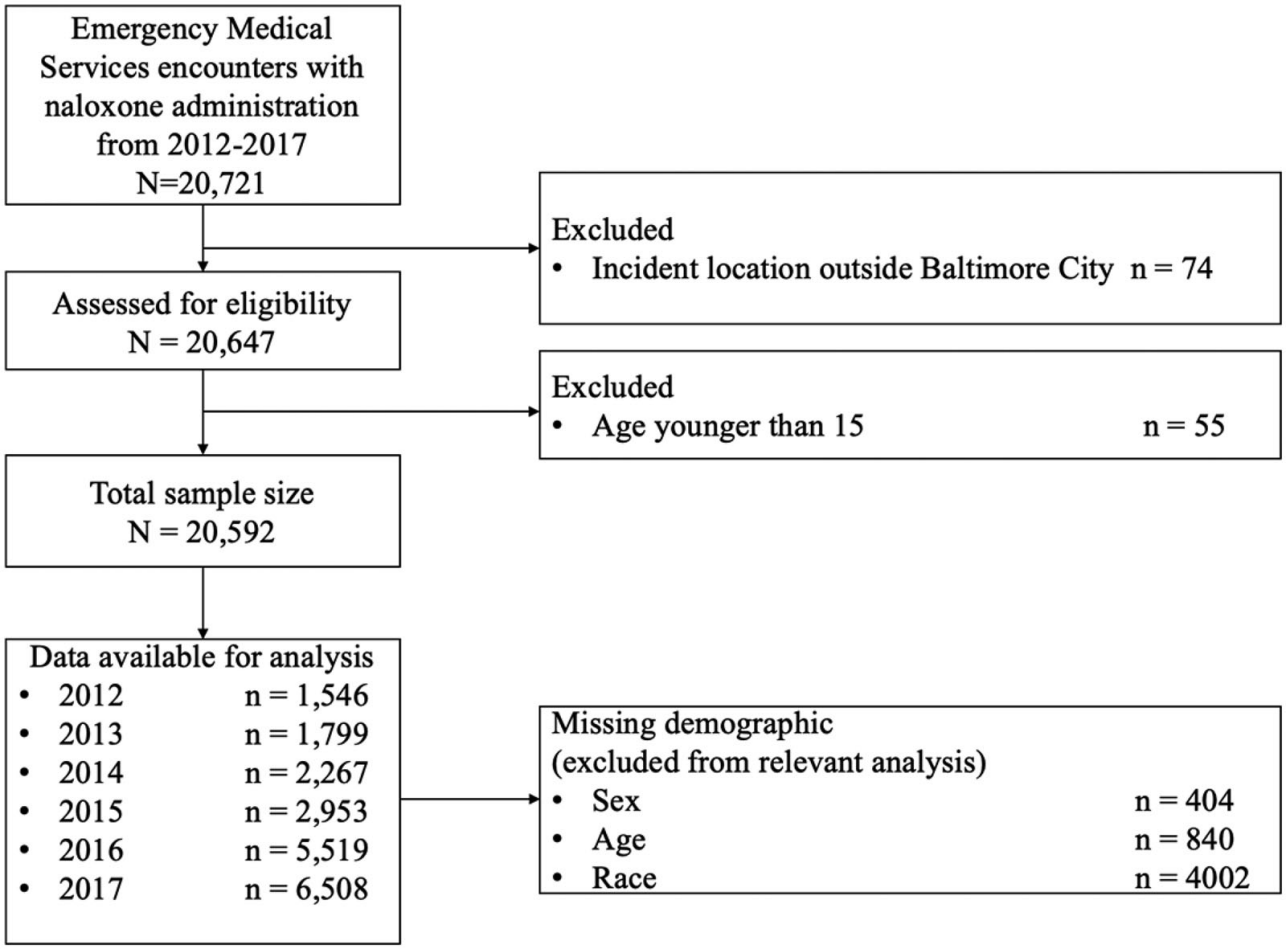
Flowchart of the breakdown of incidents included/excluded.

**Figure 2.**
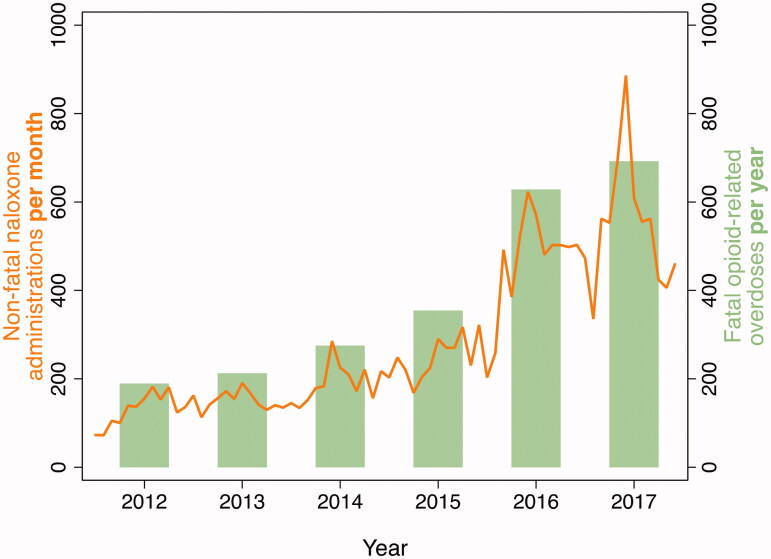
The number of EMS-related non-fatal naloxone administrations per month and number of fatal opioid-related overdoses per year within Baltimore City.

From 2012 to 2017, there were 20,592 EMS encounters with reported naloxone administration among individuals 15 years of age or older in Baltimore City (Figure 1). The number of such incidents increased every year (Figure 2 and Table 1), constituting more than a four-fold increase from 2012 (*n* = 1,546) to 2017 (*n* = 6,508). Among residents 15 years of age or older, the incidence rate increased from 3.2 (95% C.I., 1.2–8.1) per 1,000 residents in 2012 to 11.0 (95% C.I., 4.3–28.3) per 1,000 residents in 2017. The highest proportional yearly increase occurred from 2015 to 2016 (87% increase). 69.0% (*n* = 14,201) patients were reported and transported to a hospital after naloxone administration.

**Table 1.** Demographics distribution and percentages of EMS encounters with naloxone administration within Baltimore City for patients age 15 or older from 2012 to 2017.

	2012		2013		2014		2015		2016		2017		Total	
	*N*	(%)	*N*	(%)	*N*	(%)	*N*	(%)	*N*	(%)	*N*	(%)	*N*	(%)
**Total**	1,546	(7.51)	1,799	(8.74)	2,267	(11.01)	2,953	(14.34)	5,519	(26.80)	6,508	(31.60)	20,592	(100.00)
**Sex**														
Female	484	(31.31)	613	(34.07)	691	(30.48)	900	(30.48)	1,486	(26.93)	1,665	(25.58)	5,839	(28.36)
Male	1,035	(66.95)	1,123	(62.42)	1,561	(68.86)	2,034	(68.88)	3,936	(71.32)	4,660	(71.60)	14,349	(69.68)
Missing	27	(1.75)	63	(3.50)	15	(0.66)	19	(0.64)	97	(1.76)	183	(2.81)	404	(1.96)
**Race**														
White	391	(25.29)	513	(28.52)	600	(26.47)	773	(26.18)	1,386	(25.11)	1,570	(24.12)	5,233	(25.41)
African American	615	(39.78)	829	(46.08)	1,089	(48.04)	1,548	(52.42)	3,113	(56.41)	3,631	(55.79)	10,825	(52.57)
Other	41	(2.65)	38	(2.11)	53	(2.34)	81	(2.74)	134	(2.43)	185	(2.84)	532	(2.58)
Missing	499	(32.28)	419	(23.29)	525	(23.16)	551	(18.66)	886	(16.05)	1,122	(17.24)	4,002	(19.43)
**Age**														
15–19	26	(1.68)	27	(1.50)	21	(0.93)	28	(0.95)	34	(0.62)	26	(0.40)	162	(0.79)
20–24	79	(5.11)	87	(4.84)	107	(4.72)	132	(4.47)	237	(4.29)	239	(3.67)	881	(4.28)
25–29	110	(7.12)	136	(7.56)	171	(7.54)	235	(7.96)	404	(7.32)	508	(7.81)	1,564	(7.60)
30–34	99	(6.40)	155	(8.62)	165	(7.28)	214	(7.25)	409	(7.41)	525	(8.07)	1,567	(7.61)
35–39	83	(5.37)	122	(6.78)	157	(6.93)	231	(7.82)	386	(6.99)	454	(6.98)	1,433	(6.96)
40–44	145	(9.38)	172	(9.56)	227	(10.01)	253	(8.57)	407	(7.37)	448	(6.88)	1,652	(8.02)
45–49	193	(12.48)	229	(12.73)	294	(12.97)	407	(13.78)	742	(13.44)	812	(12.48)	2,677	(13.00)
50–54	230	(14.88)	298	(16.56)	375	(16.54)	492	(16.66)	870	(15.76)	1,048	(16.10)	3,313	(16.09)
55–59	164	(10.61)	222	(12.34)	293	(12.92)	395	(13.38)	901	(16.33)	1,001	(15.38)	2,976	(14.45)
60–64	105	(6.79)	133	(7.39)	192	(8.47)	221	(7.48)	458	(8.30)	554	(8.51)	1,663	(8.08)
65–69	51	(3.30)	62	(3.45)	79	(3.48)	124	(4.20)	266	(4.82)	315	(4.84)	897	(4.36)
70–74	18	(1.16)	28	(1.56)	57	(2.51)	67	(2.27)	96	(1.74)	109	(1.67)	375	(1.82)
75–79	10	(0.65)	18	(1.00)	49	(2.16)	30	(1.02)	64	(1.16)	63	(0.97)	234	(1.14)
80–84	12	(0.78)	22	(1.22)	13	(0.57)	34	(1.15)	37	(0.67)	45	(0.69)	163	(0.79)
85 or older	16	(1.03)	21	(1.17)	35	(1.54)	42	(1.42)	42	(0.76)	39	(0.60)	195	(0.95)
Missing	205	(13.26)	67	(3.72)	32	(1.41)	48	(1.63)	166	(3.01)	322	(4.95)	840	(4.08)

### Incidence rate by census tract

**Figure 3.**
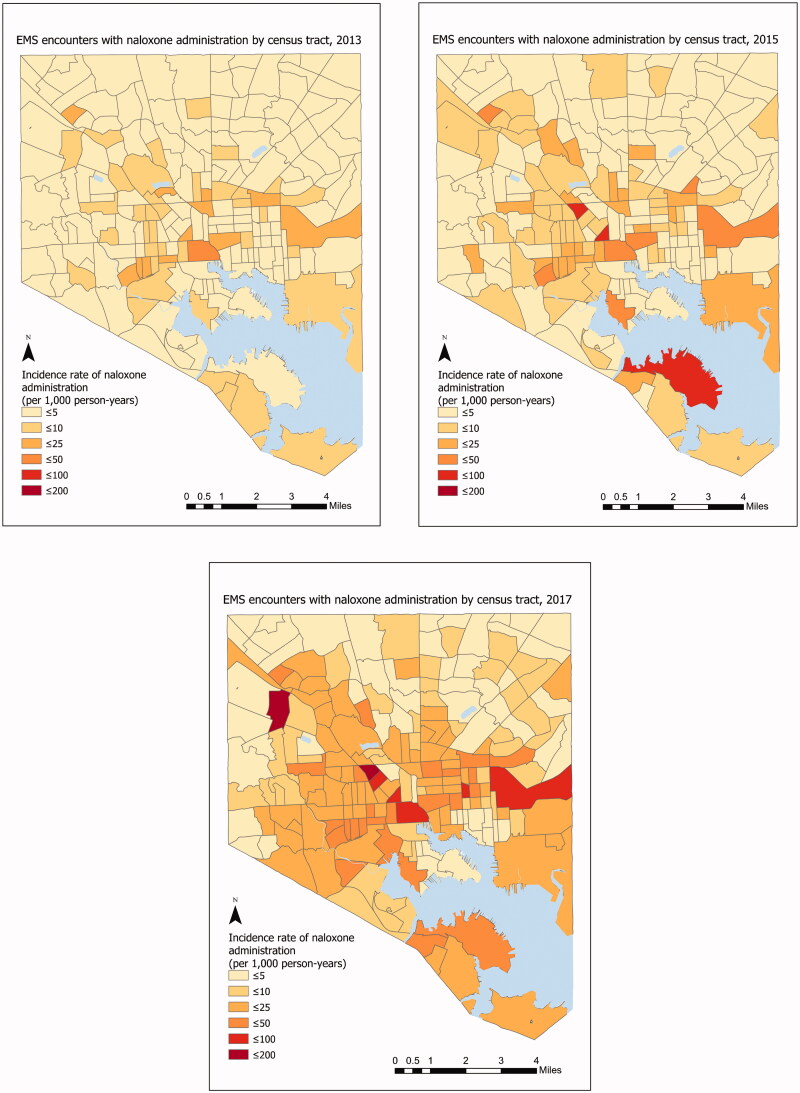
The incidence rate of EMS encounters with naloxone administration in Baltimore City by census tract, 2013, 2015 and 2017. EMS encounters among 15 years of age or older. Year-specific population estimates from Census tract [20].

Figure 3 shows the incidence rate of EMS encounters with naloxone administration per 1,000 residents for each of 200 census tracts in 2013, 2015, and 2017. In 2013, the mean incidence was 7.7 across census tracts, and in 2017 the mean incidence was 32.5 across census tracts. In 2013 there were only two census tracts (1% of all census tracts) that had more than 50 EMS incidents with naloxone administration, whereas in 2017 there were 39 census tracts (19.5% of census tracts) with more than 50 EMS incidents with naloxone administration.

### Patient demographics

**Figure 4.**
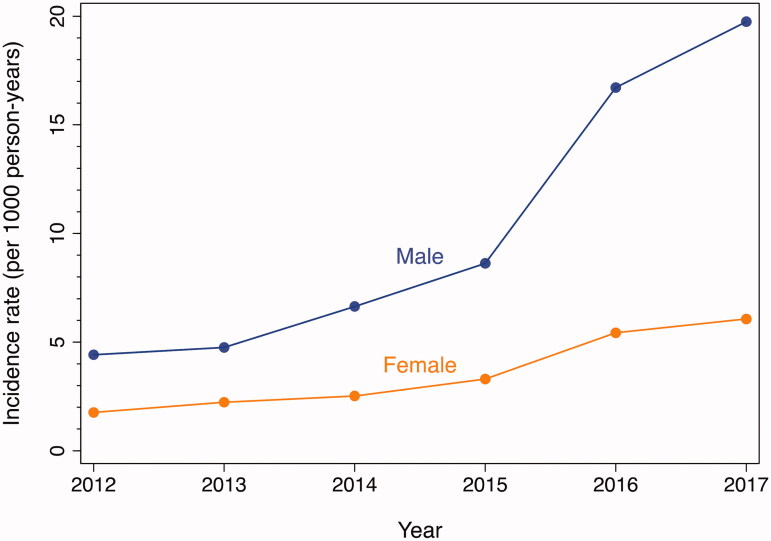
Incidence rates of EMS encounters with naloxone administration by sex, Baltimore City, 2012–2017. EMS encounters among 15 years of age or older. Year-specific population estimates from US Census [20].

The incidence rate of naloxone administration increased every year for both males and females (Figure 4 and Table 2). The incidence rate for males increased from 4.8 per 1000 residents in 2013 to 19.4 per 1000 residents in 2017. The incidence rate for females increased from 2.2 per 1000 residents in 2013 to 6.1 per 1000 residents in 2017. The incident rate ratio for 2015 vs. 2013 and 2017 vs. 2015 were significantly higher for males than females, indicating a larger proportional increase in incidence.

**Table 2.** Incidence rate (IR) of naloxone administration per 1,000 person-years and incidence rate ratios (IRR) for 2015 vs. 2013 and 2017 vs. 2015 by demographic characteristics.

	2013		2015		2017		2015 vs. 2013		2017 vs. 2015	
	IR	(95% CI)	IR	(95% CI)	IR	(95% CI)	IRR	(95% CI)	IRR	(95% CI)
Total	3.2	(1.2, 8.1)	5.4	(2.1, 13.9)	11	(4.3, 28.3)	1.6	(1.6, 1.7)	2.2	(2.1, 2.3)
Sex										
Female	2.2	(2.1, 2.4)	3.3	(3.1, 3.5)	6.1	(5.8, 6.4)	1.5	(1.3, 1.6)	1.8	(1.7, 2.0)
Male	4.8	(4.5, 5.0)	8.6	(8.3, 9.0)	19.7	(19.2, 20.3)	1.8	(1.7, 2.0)	2.3	(2.2, 2.4)
	χ^2 (1)=226.4, *p* < .001		χ^2 (1)=226.4, *p* < .001		χ^2 (1)=1713.2, *p* < .001		χ^2 (1)=10.3, *p* = .001		χ^2 (1)=20.2, *p* < .001	
Race/ethnicity										
White	3.4	(3.1, 3.7)	5.1	(4.7, 5.4)	10.3	(9.8, 10.8)	1.5	(1.3, 1.7)	2.0	(1.9, 2.2)
African American	2.6	(2.5, 2.8)	5.0	(4.7, 5.2)	11.6	(11.2, 12.0)	1.9	(1.7, 2.0)	2.3	(2.2, 2.5)
Other	0.9	(0.6, 1.2)	1.8	(1.5, 2.3)	4.2	(3.6, 4.8)	2.1	(1.4, 3.1)	2.3	(1.8, 3.0)
	χ^2 (2)=73.5, *p* < .001		χ^2 (2)=79.6, *p* < .001		χ^2 (2)=188.4, *p* < .001		χ^2 (2)=10.4, *p* = .006		χ^2 (2)=7.5, *p* = .02	
Age										
15–19	0.7	(0.5, 1.1)	0.8	(0.5, 1.1)	0.7	(0.5, 1.0)	1.0	(0.6, 1.8)	0.9	(0.5, 1.6)
20–24	1.9	(1.5, 2.3)	2.8	(2.3, 3.3)	5.0	(4.4, 5.7)	1.5	(1.1, 2.0)	1.8	(1.5, 2.2)
25–29	2.2	(1.8, 2.6)	3.7	(3.3, 4.2)	8.1	(7.4, 8.8)	1.7	(1.4, 2.1)	2.2	(1.9, 2.5)
30–34	2.9	(2.5, 3.4)	4.0	(3.5, 4.6)	9.8	(9.0, 10.7)	1.4	(1.1, 1.7)	2.4	(2.1, 2.9)
35–39	3.0	(2.5, 3.6)	5.7	(5.0, 6.5)	11.2	(10.2, 12.3)	1.9	(1.5, 2.4)	2.0	(1.7, 2.3)
40–44	5.0	(4.3, 5.8)	7.3	(6.5, 8.3)	13.0	(11.8, 14.2)	1.5	(1.2, 1.8)	1.8	(1.5, 2.1)
45–49	6.2	(5.4, 7.0)	11.0	(10.0, 12.2)	22.0	(20.5, 23.6)	1.8	(1.5, 2.1)	2.0	(1.8, 2.2)
50–54	7.1	(6.4, 8.0)	11.8	(10.8, 12.9)	25.1	(23.6, 26.7)	1.6	(1.4, 1.9)	2.1	(1.9, 2.4)
55–59	5.3	(4.6, 6.0)	9.5	(8.6, 10.5)	23.9	(22.4, 25.4)	1.8	(1.5, 2.1)	2.5	(2.2, 2.8)
60–64	3.7	(3.2, 4.4)	6.2	(5.5, 7.1)	15.7	(14.4, 17.0)	1.7	(1.3, 2.1)	2.5	(2.2, 2.9)
65–69	2.2	(1.8, 2.9)	4.5	(3.8, 5.4)	11.4	(10.2, 12.8)	2.0	(1.5, 2.7)	2.5	(2.0, 3.1)
70–74	1.5	(1.0, 2.2)	3.6	(2.8, 4.6)	5.9	(4.9, 7.1)	2.4	(1.5, 3.7)	1.6	(1.2, 2.2)
75–79	1.4	(0.9, 2.2)	2.5	(1.8, 3.5)	4.8	(3.7, 6.1)	1.8	(1.0, 3.3)	1.9	(1.3, 2.9)
80–84	2.3	(1.5, 3.5)	3.7	(2.7, 5.2)	4.8	(3.6, 6.4)	1.6	(1.0, 2.8)	1.3	(0.8, 2.0)
85 or older	2.0	(1.3, 3.1)	4.0	(3.0, 5.4)	3.7	(2.7, 5.1)	2.0	(1.2, 3.4)	0.9	(0.6, 1.4)
	χ^2 (14)=461.2, *p* < .001		χ^2 (14)=785.9, *p* < .001		χ^2 (14)=1819.4, *p* < .001		χ^2 (14)=15.9, *p* = .33		χ^2 (14)=59.7, *p* < .001	

*Chi-Square tests used for testing significant variation in each demographic characteristic group.

**Figure 5.**
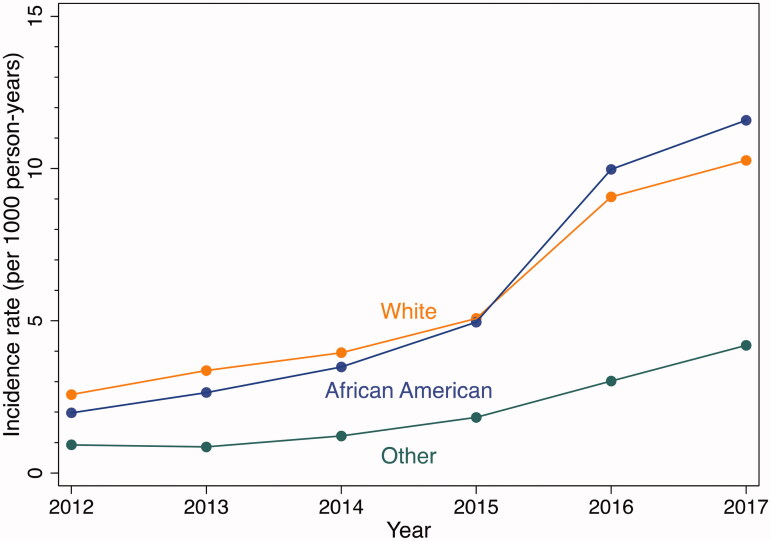
Incidence rates of EMS encounters with naloxone administration by race, Baltimore City, 2012–2017. EMS encounters among 15 years of age or older. Year-specific population estimates from US Census [20].

The incidence rate of naloxone administrations increased every year for each racial/ethnic group (Figure 5 and Table 2). Overall, the incidence was higher for whites and African Americans than for other racial/ethnic groups. Incidence rates among whites were significantly higher than among African Americans in 2013 (3.4 vs 2.6 *p* < .001) and significantly lower than among African Americans in 2017 (10.3 vs 11.6 *p* < .001). For both 2015 vs. 2013 and 2017 vs. 2015, Whites had the lowest proportional increase in incidence (2015 vs 2013 IRR: 1.5, 95% C.I., 1.3–1.7, 2017 vs 2015 IRR, 2.0, 95% C.I., 1.9, 2.2).

**Figure 6.**
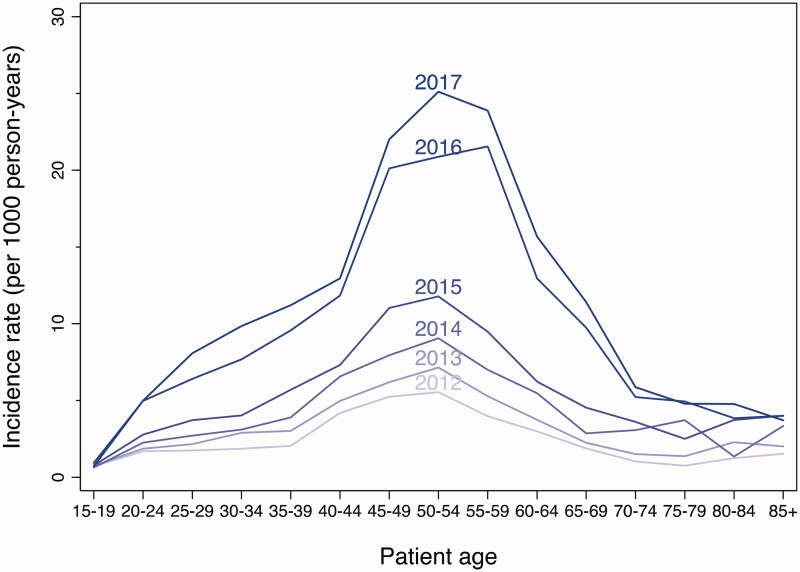
Incidence rates of EMS encounters with naloxone administration by year and age group, Baltimore City, 2012–2017. EMS encounters among 15 years of age or older. Year-specific population estimates from US Census [20].

There was significant variation in incidence across age groups, with the highest incidence generally in the 50–54 age group and lower rates among the youngest and oldest age groups (Figure 6 and Table 2). The incidence rate of naloxone administration increased every year for almost all age groups, particularly for those in the 25–64 age group. In 2013, only one age group (50–54) had an incidence rate higher than 7 per 1,000 residents, whereas, in 2017, nine age groups (ranging from 25 to 69) had an incidence rate higher than 7 per 1,000 residents. For 2015 vs. 2013, there was no significant variation in the incident rate ratios across age groups. However, for 2017 vs. 2015, there was significant variation in the incident rate ratios across age groups, with the highest proportional increases among the 25–69 age groups.

## Discussion

EMS incidents with naloxone administration increased over four-fold from 1,546 naloxone administrations in 2012 to 6,508 administrations in 2017. EMS encounters with naloxone administration followed a temporal pattern similar to fatal opioid-related overdoses in Baltimore City at a similar time (Figure 2), based on the fatal overdose date from the Maryland Department of Health [25]. The city-wide pattern in the increase in EMS encounters with naloxone administration was mirrored in almost all groups based on sex, race/ethnicity, age, and geography. This suggests that changes in factors that have increased opioid-related overdose risk in Baltimore may be widely distributed, and an increase in the adulteration of the illicit drug supply with fentanyl-related compounds would not be inconsistent with the epidemiology.

Despite the consistency of the overall pattern, there are some important variations. Throughout the study period, the incidence was higher among males than females, higher among non-Hispanic whites and non-Hispanic African Americans than those of other races/ethnicity, and higher among the middle age groups (25–69) than younger or older age groups. Regarding changes in incidence over time, incidence rate ratios for both 2015 vs. 2013 and 2017 vs. 2015 were significantly higher for males than females, African Americans and those of other race/ethnicity than whites, and those of the middle age groups than those younger or older.

Another important consideration is that while the proportional increases in population-based incidence over time tended to be more similar than dissimilar across demographic groups, the similar proportional increases often belie large differences in absolute increases. For example, while the proportional increase in incidence from 2015 to 2017 was similar for African Americans and those of other races/ethnicity (IRR = 2.3 for both), the number of EMS runs with naloxone administration increased by 6.6 per 1000 for African Americans compared to only 2.4 per 1000 for other race/ethnicity. This difference between proportional and absolute increases demonstrates that groups with an already high burden of an overdose in 2015 (or 2013) are also the groups experiencing the largest absolute increases in burden.

### The value of EMS for overdose epidemiology and prevention

A previous study concluded that opioid-related EMS data could serve as early detection of trends in opioid overdose and lead to harm reduction [26]. Patterns of EMS encounters with naloxone administration appear to be an excellent proxy for patterns of opioid-related overdoses based on the consistency of fatal overdose rates over time (Figure 2). The former also has the advantages of close to real-time data availability, geographic and temporal precision, and opportunities for prevention. Prior overdose is one of the strongest predictors of fatal overdose. We found that the number of EMS encounters with naloxone administrations was approximately twelve-fold higher than fatal opioid-related overdoses for each calendar year. Compared to mortality data, EMS data may reveal emerging trends more quickly and allow for a more granular understanding of opioid overdose epidemiology and prevention.

EMS plays a central role in preventing fatal opioid-related overdoses through the administration of naloxone, provision of other emergency services, and transportation to medical facilities. There does not appear to be a good point estimate in the literature of the effectiveness of EMS naloxone administration in preventing fatal overdoses [27]; however, given the number of EMS encounters with naloxone administration (20,592) and the percent with subsequent improvement in patient status (79%), the number of fatal opioid-related overdoses averted is likely substantial. The pivotal role of EMS could be expanded or supplemented. EMS encounters with naloxone administration may be suitable for naloxone distribution and other harm reduction interventions. Individuals who use opioids encountered through EMS could be followed up by social workers or peer navigators to link them with needed medical and social services, including naloxone distribution and drug treatment services. More general epidemiologic data could be used to identify higher-risk geographic areas for targeted “bad batch” alerts [28], naloxone distribution, and other harm reduction services.

EMS encounters with naloxone administration could also be used to evaluate the impact of overdose prevention interventions and public health services. At the person level, EMS data could be used to determine whether the provision of naloxone kits or other services is associated with a reduced risk of subsequent EMS encounters with naloxone administration. At the neighbourhood level, EMS data could be used in space-time count models to evaluate whether interventions are associated with lower than expected counts of EMS encounters with naloxone administration. Knowing EMS data as a beneficial surveillance tool for harm reduction, local stakeholders should closely work and highly engage with research teams for future studies to maximise the data and resource utilisation efficiency. With the growing use of EMS for surveillance, there is an urgent need for researchers and epidemiologists to engage with community partners. The role data may play in spurring action on combating the opioid epidemic.

### Limitations

This study had several limitations. First, although conditions improved for the majority of patients following the administration of naloxone (79%), some patients administered naloxone may not have been suffering from an opioid-related overdose. Relatedly, there may be some patients experiencing opioid-related overdose who were not administered naloxone and therefore not reflected in these analyses. Consequently, there may be misclassification of opioid-related overdoses based on naloxone administration.

In addition to misclassification among individuals with EMS records, there are also opioid-related overdoses outside of the EMS system. Naloxone is available to all adults in Baltimore City without a prescription, and naloxone may be administered outside of the health care system by bystanders if it is readily available. In other cases, individuals who have overdosed may be transported to emergency departments independent of EMS.

Second, EMS records may be prone to the missingness and misclassification of patient demographics. Missing data on race and other demographics may be a frequent concern with EMS data. For example, Zozula et al. found that 40% of EMS encounters with naloxone administration in Cincinnati were missing data on race [20]. In our data, 19% of patients were missing a racial identification. Demographic factors may not be relevant to the provision of emergency medical care, and the altered mental status of some patients may make it difficult for them to self-report age, race, ethnicity, and sex. Anecdotally, EMS staff reported that demographic data were often based on medick impression rather than on self-report because patients may not be able to self-report demographics or because demographics may be viewed as of little importance during an emergency situation. Differences in how race and ethnicity are determined on the census versus in the EMS records could account for the lower population-based rate of naloxone administration among those in the “other” race/ethnicity category. Individuals who might not consider themselves to be either White or Black may be incorrectly categorised as such by EMS providers. Similarly, while the census allows for individuals to specify more than one racial category, that was not possible with the EMS records. Consequently, those in the “other” race/ethnicity category may be underrepresented in the EMS records, artificially lowering the population-based incidence.

Lastly, our study did not extend beyond demographic, geographic, and temporal factors associated with EMS encounters with naloxone administration. Many factors drive opioid-related overdose, including drug using practices, characteristics of neighbourhoods, characteristics of the drug supply, and naloxone availability, to name a few. The results of the present study should be considered in the context of other studies that more fully elucidate the drivers of opioid-related overdose in Baltimore and elsewhere.

## Conclusions

EMS records can be a valuable tool in responding to the opioid-related overdose epidemic. The findings illustrate the scale of the epidemic in Baltimore, with 11 naloxone administrations per 1000 adults in 2017. With more than a four-fold city-wide increase from 2012 to 2017, EMS data may also demonstrate the urgency of public health response, but the specificity of EMS records in regard to demographics and geography can also be used to develop targeted responses for individuals and groups at highest risk of non-fatal and fatal overdose.

## Data Availability

Data generated at a central, large-scale facility, available upon request.

## References

[1] Mattson CL, Tanz LJ, Quinn K, et al. Trends and geographic patterns in drug and synthetic opioid overdose deaths — United States. MMWR Morb Mortal Wkly Rep. 2021;70(6):202–2019. 33571180 10.15585/mmwr.mm7006a4 PMC7877587

[2] Centers for Disease Control and Prevention. Drug overdose deaths in the U.S. top 100,000 annually. https://www.cdc.gov/nchs/pressroom/nchs_press_releases/2021/20211117.htm.

[3] CDC WONDER. [cited 2021 Sept 7]. Available from: https://wonder.cdc.gov/.

[4] Jones CM, Einstein EB, Compton WM. Changes in synthetic opioid involvement in drug overdose deaths in the United States, 2010–2016. JAMA. 2018;319(17):1819–1821. 29715347 10.1001/jama.2018.2844 PMC6583033

[5] Maryland Department of Health. unintentional drug- and alcohol-related intoxication deaths in Maryland, 2019. Available from: 2020. https://health.maryland.gov/vsa/Documents/Overdose/Annual_2019_Drug_Intox_Report.pdf.

[6] Cepeda MS, Fife D, Chow W, et al. Assessing opioid shopping behaviour: a large cohort study from a medication dispensing database in the US. Drug Saf. 2012;35(4):325–334. 22339505 10.2165/11596600-000000000-00000

[7] Hall AJ, Logan JE, Toblin RL, et al. Patterns of abuse among unintentional pharmaceutical overdose fatalities. JAMA. 2008;300(22):2613–2620. 19066381 10.1001/jama.2008.802

[8] Hirshon JM, Warner M, Irvin CB, et al. Research using emergency department–related data sets: current status and future directions. Acad Emerg Med. 2009;16(11):1103–1109. 20053229 10.1111/j.1553-2712.2009.00554.x PMC3744773

[9] Maragh-Bass AC, Fields JC, McWilliams J, et al. Challenges and opportunities to engaging emergency medical service providers in substance use research: a qualitative study. Prehosp Disaster Med. 2017;32(2):148–155. 28122657 10.1017/S1049023X16001424

[10] Knowlton A, Weir BW, Hazzard F, et al. EMS runs for suspected opioid overdose: implications for surveillance and prevention. Prehosp Emerg Care. 2013;17(3):317–329. 23734988 10.3109/10903127.2013.792888 PMC3682796

[11] Schneider MF, Bailey JE, Cicero TJ, et al. Integrating nine prescription opioid analgesics and/or four signal detection systems to summarize statewide prescription drug abuse in the United States in 2007. Pharmacoepidemiol Drug Saf. 2009;18(9):778–790. 19536784 10.1002/pds.1780

[12] NVSS – National Vital Statistics System Homepage. [updated 2021 July 30; cited 2021 September 7]. Availble from: https://www.cdc.gov/nchs/nvss/index.htm.

[13] Products – Data Briefs – Number 294 – December 2017. [updated 2019 June 6; cited 2021 Sept 7]. Available from: https://www.cdc.gov/nchs/products/databriefs/db294.htm.

[14] Banta-Green CJ, Coffin PO, Schoeppe JA, et al. Heroin and pharmaceutical opioid overdose events: emergency medical response characteristics. Drug Alcohol Depend. 2017;178:1–6. 28623805 10.1016/j.drugalcdep.2017.04.021

[15] Belz D, Lieb J, Rea T, et al. Naloxone use in a tiered-response emergency medical services system. Prehosp Emerg Care. 2006;10(4):468–471. 16997776 10.1080/10903120600885134

[16] Council of State and Territorial Epidemiologists. Nonfatal opioid overdose standardized surveillance case definition; 2018.

[17] Merchant RC, Schwartzapfel BL, Wolf FA, et al. Demographic, geographic, and temporal patterns of ambulance runs for suspected opiate overdose in Rhode Island, 1997–20021. Subst Use Misuse. 2006;41(9):1209–1226. 16861173 10.1080/10826080600751898

[18] Madah-Amiri D, Clausen T, Myrmel L, et al. Circumstances surrounding non-fatal opioid overdoses attended by ambulance services. Drug Alcohol Rev. 2017;36(3):288–294. 28036135 10.1111/dar.12451 PMC5434850

[19] Knowlton A, Weir BW, Hughes BS, et al. Patient demographic and health factors associated with frequent use of emergency medical services in a mid-sized city. Acad Emerg Med. 2013;20(11):1101–1111. 24238312 10.1111/acem.12253 PMC4063348

[20] Zozula A, Neth MR, Hogan AN, et al. Non-transport after prehospital naloxone administration is associated with higher risk of subsequent non-fatal overdose. Prehosp Emerg Care. 2022;26(2):272–279. 33535012 10.1080/10903127.2021.1884324

[21] U.S. Census Bureau. 2010 TIGER/Line® Shapefiles Technical Documentation; 2010.

[22] Esri Inc. ArcGIS Pro; 2020. https://www.esri.com/en-us/arcgis/products/arcgis-pro/overview.

[23] U.S. Census Bureau QuickFacts: Baltimore City, Maryland; United States. [cited 2021 Sept 7]. Available from: https://www.census.gov/quickfacts/fact/table/baltimorecitymaryland,U. S/PST045219.

[24] Baltimore City’s Response to the Opioid Epidemic | Baltimore City Health Department. [cited 2021 Sept 7]. Available from: https://health.baltimorecity.gov/opioid-overdose/baltimore-city-overdose-prevention-and-response-information.

[25] Annual_2018_Drug_Intox_Report.pdf. [cited 2021 Oct 4]. Available from: https://health.maryland.gov/bha/Documents/Annual_2018_Drug_Intox_Report.pdf.

[26] Khare A, Sidana A, Mohemmed A, et al. Acceleration of opioid-related EMS runs in the spring of 2020: the national emergency medical services information system data for 2018–2020. Drug Alcohol Depend. 2022;232:109271. 35051696 10.1016/j.drugalcdep.2022.109271

[27] Grover JM, Alabdrabalnabi T, Patel MD, et al. Measuring a crisis: questioning the use of naloxone administrations as a marker for opioid overdoses in a large U.S. EMS system. Prehosp Emerg Care. 2018;22(3):281–289. 29297739 10.1080/10903127.2017.1387628

[28] Bad Batch Alert. Bad Batch Alert. [cited 2021 Oct 22]. Available from: http://www.badbatchalert.com/.

